# Anisotropy of Co^II^ transferred to the Cr_7_Co polymetallic cluster *via* strong exchange interactions†
†Electronic supplementary information (ESI) available: Details of the experimental and theoretical procedures used and supplementary results. CCDC 1810773. For ESI and crystallographic data in CIF or other electronic format see DOI: 10.1039/c8sc00163d


**DOI:** 10.1039/c8sc00163d

**Published:** 2018-03-07

**Authors:** Elena Garlatti, Tatiana Guidi, Alessandro Chiesa, Simon Ansbro, Michael L. Baker, Jacques Ollivier, Hannu Mutka, Grigore A. Timco, Inigo Vitorica-Yrezabal, Eva Pavarini, Paolo Santini, Giuseppe Amoretti, Richard E. P. Winpenny, Stefano Carretta

**Affiliations:** a Dipartimento di Scienze Matematiche, Fisiche e Informatiche , Università di Parma , I-43124 Parma , Italy . Email: stefano.carretta@unipr.it; b ISIS Facility , Rutherford Appleton Laboratory , OX11 0QX Didcot , UK; c Institute for Advanced Simulation , Forschungszentrum Jülich , 52425 Jülich , Germany; d The School of Chemistry , Photon Science Institute , The University of Manchester , M13 9PL Manchester , UK; e Institut Laue-Langevin , 71 Avenue des Martyrs CS 20156 , Grenoble Cedex 9 F-38042 , France; f The School of Chemistry , The University of Manchester at Harwell , Didcot , OX11 0FA , UK; g JARA High-Performance Computing , RWTH Aachen University , 52062 Aachen , Germany

## Abstract

The Cr_7_Co ring represents a model system to understand how the anisotropy of a Co^II^ ion is transferred to the effective anisotropy of a polymetallic cluster by strong exchange interactions. Combining sizeable anisotropy with exchange interactions is an important point in the understanding and design of new anisotropic molecular nanomagnets addressing fundamental and applicative issues. By combining electron paramagnetic resonance and inelastic neutron scattering measurements with spin Hamiltonian and *ab initio* calculations, we have investigated in detail the anisotropy of the Co^II^ ion embedded in the antiferromagnetic ring. Our results demonstrate a strong and anisotropic exchange interaction between the Co and the neighbouring Cr ions, which effectively transmits the anisotropy to the whole molecule.

## Abstract 2

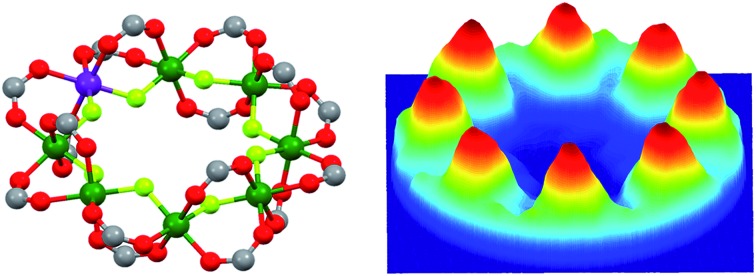
In the Cr_7_Co model-system the anisotropy of Co^II^ is effectively transferred to the whole cluster through strong and anisotropic exchange interactions.

## 1 Introduction

Single-Molecule Magnets (SMMs) are the first generation of molecular nanomagnets and have been considered as possible means to store information in single molecules since the early 1990s.^1^,^2^ Indeed, the combination of a large magnetic moment and a strong easy-axis magnetic anisotropy creates in these systems a double-well potential with an energy barrier for the reorientation of the magnetization, resulting in magnetic bistability for the single molecule below the so-called “blocking temperature”. Thus, increasing the temperature range where molecules display SMM behaviour, in order to build miniaturized memory units, is one of the main goals of the current research in molecular magnetism. Originally this was sought in exchange-coupled 3d-metal cage complexes, by exploiting high-spin ions like Fe^III^, Mn^II^ or Cr^III^ to increase the total-spin of the molecular ground state.^3^–^7^ However, the quenching of the orbital momentum and the resulting small single-ion anisotropy have strongly limited the maximum achievable barriers. Recently it has been shown that monometallic complexes based on 3d transition metal ions can exhibit appreciably enhanced magnetic anisotropy with certain axial coordination environments that minimize Jahn–Teller distortions, leading to an almost unquenched orbital momentum.^8^ The second attempt has been to use the large anisotropy of rare-earth ions. Indeed, after the discovery of SMM behaviour in mononuclear Tb^III^ and Dy^III^ complexes,^9^,^10^ much focus has been on the design of highly-axial environments for single lanthanide ions, with the aim to reduce the efficiency of the tunnelling mechanism and to increase the blocking temperature of the molecule.^11^–^15^ This has led to a remarkable recent report of a Dy^III^ sandwich complex that retains magnetization to 60 K.^16^ These advances with mononuclear lanthanide-based SMMs are important but distract from one crucial issue that has only been partially addressed, that is understanding magnetic exchange interactions in molecules containing highly anisotropic ions. This is important not only in dinuclear Ln^III^ complexes, where the SMM behaviour results from the interplay between ligand fields and exchange interactions,^17^,^18^ but also in more diverse areas ranging from quantum information to biology. For example, highly anisotropic Fe^II^ centers are found in the active sites of proteins.^19^ Thus, we have focused our attention towards polymetallic complexes containing Co^II^, as test systems to investigate anisotropic exchange interactions. Indeed, Co^II^ in the proper coordination environment can be highly anisotropic thanks to its non-quenched orbital moment.

SMMs containing Co^II^ ions have been largely investigated in the last years, thanks to their potential for the production of very large magnetic anisotropies and thus higher blocking temperatures.^20^–^22^ However, the interpretation of magnetic data for polynuclear complexes containing Co^II^ centres is often difficult, due to their important orbital contribution. In particular, there is now a literature on Co^II^-based SMMs, but the precise nature of the magnetic exchange in these systems is still a crucial open question that need to be investigated.^20^ There have been several papers studying homometallic complexes including Co^II^^23^,^24^ and lanthanides,^25^ where exchange interactions have been investigated with different spectroscopic techniques, but studies where a highly anisotropic ion is included in a larger polymetallic complex remain rare. Anisotropic ions (such as Co^II^) coupled with other magnetic ions are important building blocks to implement efficient and scalable quantum information schemes.^26^ Moreover, this kind of compounds is expected to display rich physics associated with the unquenched orbital degrees of freedom. For instance, in antiferromagnetic highly anisotropic ring-like clusters the low-frequency dynamics should be characterized by Néel Vector Tunneling.^27^–^32^ Thus, it is important to understand in detail the anisotropic exchange interactions involving a Co^II^ ion embedded in a large polymetallic cluster.

In this work we exploit the octagonal heterometallic ring [NH_2_Me_2_][Cr_7_CoF_8_(O_2_CC^*t*^Bu)_16_] (hereafter Cr_7_Co ring) as a model system to understand how the insertion of an anisotropic Co ion strongly coupled to the Cr ones affects the anisotropy of the whole molecule. Cr–Cr exchange interactions and the Cr zero-field splittings have been already determined in the isostructural Cr_8_ and Cr_7_M (M = Ni, Mn, Zn) compounds.^32^–^35^ Thus, the combination of Electron Paramagnetic Resonance (EPR) and Inelastic Neutron Scattering (INS) techniques with *ab initio* and spin Hamiltonian calculations,^36^ allows us to investigate in detail the anisotropy of the Co^II^ ion embedded in the antiferromagnetic ring and the Cr–Co anisotropic exchange interactions. By combining EPR measurement on the isotructural Ga_7_Co compound with *ab initio* calculations, we demonstrate that the Co^II^ ion in this environment is highly anisotropic and we are able to determine the spectroscopic splitting tensor **g**_Co_. We have then performed INS measurements on both powder samples of Cr_7_Co at different temperatures and on single-crystal samples with different applied magnetic fields. Neutron scattering intensities on single-crystal samples have also been measured as a function of energy transfer and neutron momentum transfer (*Q*_*x*_, *Q*_*y*_, *Q*_*z*_) with the 4-dimensional INS (4D-INS) technique.^32^,^37^ Guided by *ab initio* calculations based on the density-functional theory + many-body approach (DFT + MB),^36^ and thanks to the thorough set of INS measurements described above, we determine the Cr–Co isotropic coupling *J* and anisotropic exchange tensor **D**. In agreement with *ab initio* calculations, we find that Cr_7_Co is characterised by a strong and highly anisotropic Cr–Co exchange interaction, which effectively transmits the anisotropy of Co^II^ to the whole molecule. As discussed in the last part of the paper, these results suggest a promising way to reach the strong coupling regime between photons and individual molecules,^38^ a crucial step for building a scalable molecule-based quantum information architecture.

## 2 Results

### 2.1 Synthesis and structural considerations

Cr_7_Co was made by the method described in ref. 39 with minor modifications detailed in the ESI.† Crystals large enough for INS studies were grown by evaporation of a solution of Cr_7_Co dissolved in Et_2_O/MeCN.

**Fig. 1:**
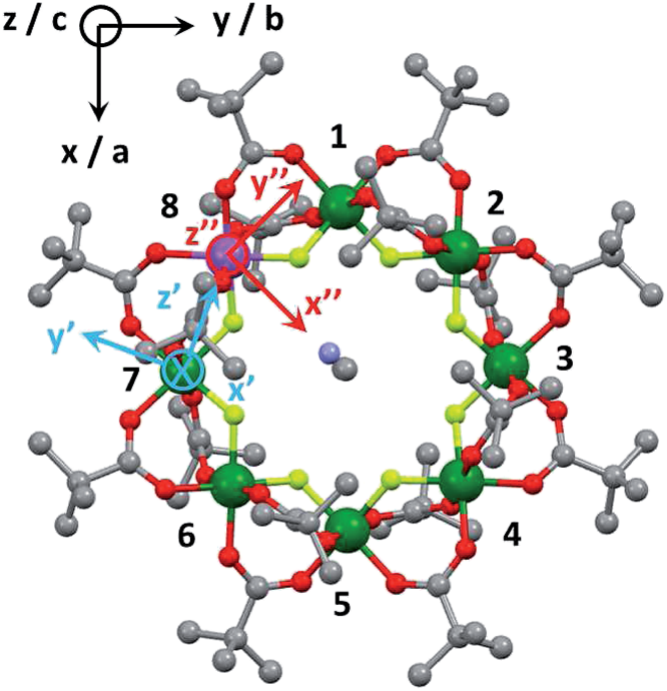
Laboratory/unit cell reference frame for the Cr_7_Co ring (black) and local reference frames of the anisotropic exchange tensor **D**_local_ (light blue) for the *s*_Co_ –*s*_7_ interaction and of the spectroscopic splitting tensor **g**_local_ (red), assuming the Co ion on site 8.

The structure of Cr_7_Co is an octagon of metals bridged on each M···M edge by one fluoride and two pivalate ligands (Fig. 1). Therefore each metal is six-coordinate, with a F_2_O_4_ coordination sphere. At the centre of the ring is a dimethylammonium cation where the protonated N atom is involved in hydrogen-bonding to bridging fluorides. Using this cation and solvents the Cr_7_Co ring crystallises in a tetragonal crystal system with a four-fold rotation axis passing through the N-atom of the cation. This four-fold axis leads to the Co^II^ ion being disordered about the eight metal sites of the ring, and to disorder of the cation.

### 2.2 Spin Hamiltonian model

The Cr_7_Co ring contains seven isotropic Cr^III^ ions, behaving as pure spin *s*_*i*_ = 3/2, and one anisotropic Co^II^ ion, that can be described at low temperatures as an effective spin *s*_Co_ = 1/2, with highly anisotropic **g** tensor and exchange interactions^40^ (see the following Section). The magnetic properties of Cr_7_Co can therefore be described by the following spin Hamiltonian (with the Co ion on site 8):1
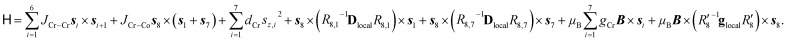



The first two terms in eqn (1) describe the dominant isotropic exchange interactions, while the third term corresponds to the axial single-ion zero-field-splitting term acting on Cr ions (with the *z*-axis perpendicular to the plane of the ring). The fourth and fifth terms take into account the anisotropic exchange interaction of the Co^II^ ion with the two neighbouring Cr ions (labelled as *s*_1_ and *s*_7_ respectively), which is described by the traceless diagonal tensor **D**_local_, referred to its local principal axis on each Cr–Co bond, with diagonal elements *D*_*yy*_, *D*_*zz*_ and *D*_*xx*_ = –(*D*_*yy*_ + *D*_*zz*_). The last terms in eqn (1) describe the Zeeman interaction with an applied magnetic field, where the spectroscopic splitting tensor is assumed isotropic for Cr^III^ (*g*_Cr_ = 1.98) and anisotropic for the Co^II^ ion (**g**_local_). The local principal axis for both **D**_local_ and **g**_local_ tensors have been determined by the DFT + MB approach described below and are depicted in Fig. 1. The matrices *R*_8,1–7_ and *R*′_8_ in eqn (1) transform the **D**_local_ and **g**_local_ tensors from their local reference frames to the laboratory/unit cell reference system (see Fig. 1). At last, the delocalization of the Co^II^ ion along the ring in the crystal (equal probability on each site) is taken into account in modeling the experimental results. Cr–Cr exchange interactions and Cr zero-field splittings have already been determined in the isostructural Cr_8_ and Cr_7_M (M = Ni, Mn, Zn) compounds, yielding *J*_Cr–Cr_ = 16.9 K and *d*_Cr_ = –0.44 K.^33^–^35^ Hence, here we can focus on Co^II^ and Cr–Co interactions to extract *J*_Cr–Co_, **D**_local_ and **g**_local_ by combining EPR and INS data with the spin Hamiltonian and *ab initio* calculations.

### 2.3 *Ab initio* DFT + MB results

*Ab initio* calculations based on the DFT + MB approach described in ref. 36 have been used as a guide for fitting the spin Hamiltonian parameters. In contrast with conventional DFT approaches, relying on approximations of the exchange-correlation functional (the most popular being LDA/GGA or their extensions, such as the hybrid B3LYP), here strong electron–electron correlations are explicitly included in the description of the low energy 3d electrons. This is achieved by constructing a system specific generalized Hubbard model (see details in the ESI†), explicitely including a Coulomb tensor, as well as inter-site electron hopping, crystal field splittings and spin orbit effects. The parameters of this model are deduced *ab initio*, by means of DFT based calculations either in the local-density (one-electron terms) or in the constrained-local-density approximations (Coulomb tensor). In a second step, the spin Hamiltonian is obtained by a canonical transformation of the Hubbard model, without any *a priori* assumption on its form.

This method ensures an accurate description of strongly correlated systems (such as MNMs), as shown also by a recent study of a family of Cr_7_M compounds isostructural to Cr_7_Co.^35^ Co^II^ (3d^7^ electronic configuration, *S* = 3/2) is embedded in a distorted octahedral cage of ligands (O and F). In perfect octahedral symmetry, spin–orbit coupling would split the twelve-fold degenerate (*l* = 1, *S* = 3/2) ground multiplet into a doublet, a quartet and a sextet.^40^ Here the distorted octahedral environment leads to a sequence of Kramers doublets, the lowest two being separated by about 180 K according to *ab initio* calculations (see details in the ESI†). Thus, at low temperatures we can restrict to the lowest-energy doublet and describe it as an effective spin 1/2. In this subspace, we obtain the spin Hamiltonian in eqn (1), with the principal axes of the **D**_local_ and **g**_local_ tensors sketched in Fig. 1. Our first-principles calculations predict an isotropic Cr–Co exchange constant of *J*_Cr–Co_ = 21 K and anisotropic exchange with *D*_*zz*_ = –11.2 K and *D*_*yy*_ = 10.4 K leading to (in K):2
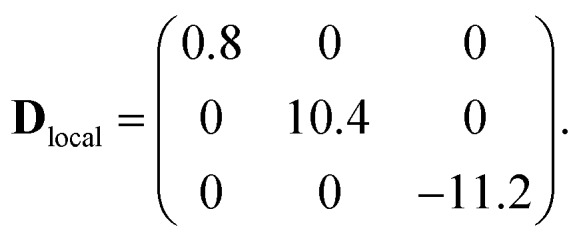



These results show two directions where the Cr–Co anisotropic exchange is strong but opposite in sign and a third direction where it is almost one order of magnitude weaker. Finally, we obtain **g**_local_ = (5.4, 3.3, 2.9). These parameters have been used as a guide for the final determination of the spin Hamiltonian based on EPR and INS data. DFT + MB calculations also indicate the presence of a significant Dzialoshinski–Moriya (D–M) interaction. However, here this interaction acts only at the second-order through the S-mixing^41^ and its effects are much weaker than those of anisotropic exchange. Thus, in the following we only consider the isotropic and anisotropic parts of the exchange interaction to keep the model with the minimum number of parameters.

### 2.4 EPR and INS spectroscopy

**Fig. 2:**
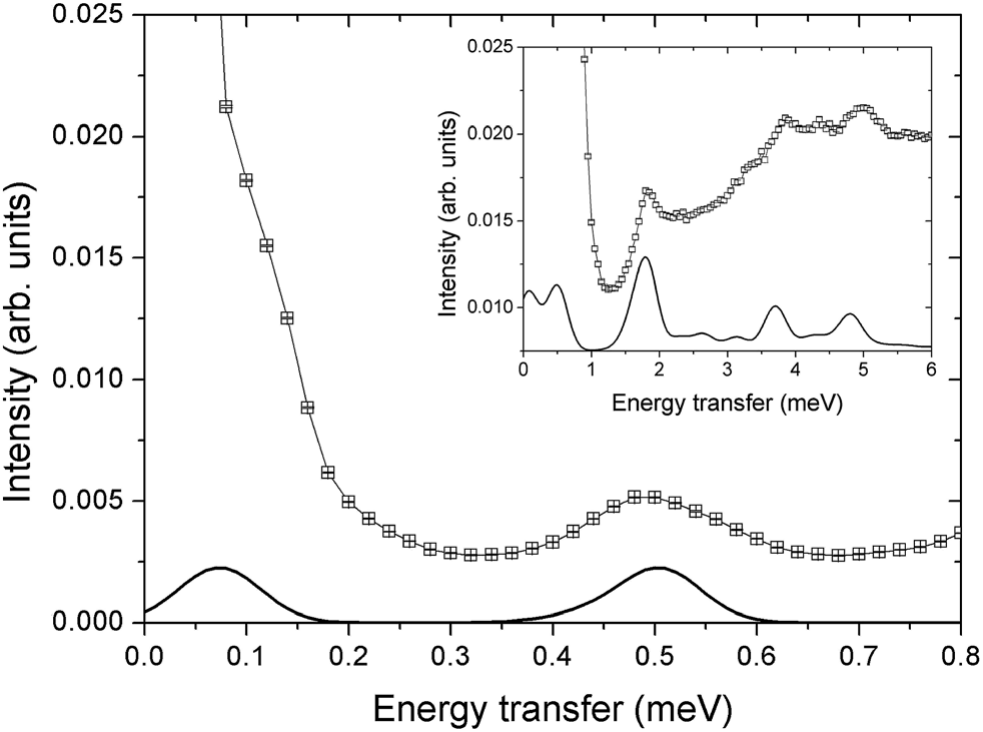
INS spectra for a Cr_7_Co single crystal collected on the IN5 spectrometer at *T* = 1.5 K with an incident neutron energy *E*_i_ = 1.9 meV (*E*_i_ = 8 meV in the inset) and integrated over the full measured Q range (black squares). The *c* axis of the single crystal is perpendicular to the horizontal scattering plane. Solid lines are simulations based on the spin Hamiltonian in eqn (1) with *J*_Cr–Co_ = 19 K and the local anisotropic exchange tensor obtained from the fitting of all the INS data. The delocalization of the Co ion along the ring is also taken into account in the simulations.

**Fig. 3:**
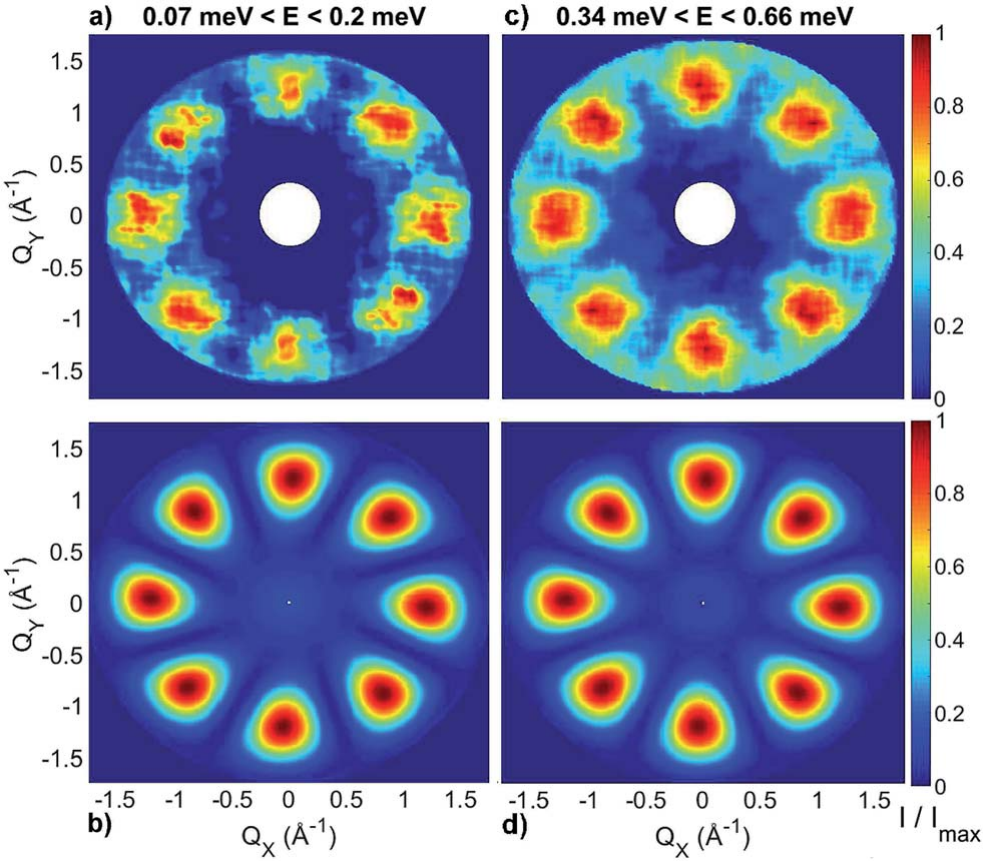
Constant-energy plots of the neutron scattering intensity measured on IN5 with an incident energy of 1.9 meV and with a sample temperature of 1.5 K. (a) and (c) Show the experimental dependency of the neutron scattering intensity of the inelastic excitations observed at 0.1 meV, 0.5 meV respectively on the two horizontal wavevector components *Q*_*x*_ – *Q*_*y*_, integrated over the full experimental *Q*_*z*_ data range (–0.2 to 0.2 Å). (b) and (d) Show the corresponding calculations based on the spin Hamiltonian in eqn (1) with *J*_Cr–Co_ = 19 K and the local anisotropic exchange tensor obtained from the fitting of all the INS data. The delocalization of the Co ion along the ring is also taken into account in the simulations. The cross-section has been integrated over energy ranges centred around the observed transition energies: 0.07 meV < *E* < 0.2 meV for (a, b) and 0.34 meV < *E* < 0.66 meV for (c, d). The colour bar reports the transition intensity normalized for the maximum in each panel.

EPR measurements have been performed on powder samples of the isotructural compound Ga_7_Co (see the ESI†). Since Ga^III^ is diamagnetic, these measurements have allowed us to selectively target the Co^II^ ion embedded in the same environment as in the Cr_7_Co ring. In Fig. S2 of the ESI† we report Q- and W-band EPR spectra, whose main features can be reproduced by assuming a very anisotropic **g**_local_ for Co^II^, with principal values (6.8, 2.9, 2.7). The results obtained with the DFT + MB method are in good agreement with the EPR findings and are used to determine the principal axis of the spectroscopic splitting tensor for the Co^II^ ion, reported in Fig. 1, yielding 
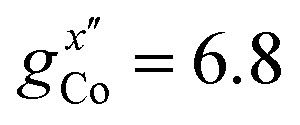
, 
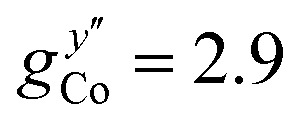
 and 
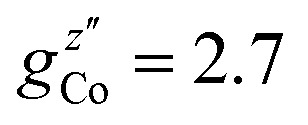
. These orientations are confirmed by the good agreement with single-crystal INS measurements in applied fields (see below). As a first step to investigate the Cr_7_Co ring, we have surveyed the full energy spectrum of a non-deuterated powder sample on the time-of-flight spectrometer IN5 at the Institute Laue Langevin in Grenoble.^42^ Measurements have been performed at three different temperatures, *T* = 1.5, 6, 15 K, with two different incident neutrons energies *E*_i_, 1.9 and 8 meV (see Fig. S3 in the ESI†). The high-resolution/low-energy-transfer spectra show two cold transitions around 0.5 meV, whereas higher-energy data show magnetic excitations up to 5 meV, with three cold peaks at 1.8, 3.7 and 4.8 meV. The same measurements at *T* = 1.5 K have also been performed on a single-crystal sample of Cr_7_Co, where we have exploited the position-sensitive detectors of IN5 to implement the four-dimensional inelastic neutron scattering (4D-INS) technique.^32^ Thanks to this technique it is possible to measure the INS cross-section not only as a function of energy, but also as a function of the three component of the momentum transfer vector **Q** (see also the ESI†). The information accessible with this technique enables us to characterize the eigenstates of the system involved in the detected excitations.^43^ INS single-crystal spectra as a function of the energy-transfer, integrated over the full **Q**-space are reported in Fig. 2 and show the same magnetic transitions measured on the powder sample. In this configuration we have been able to obtain one crucial additional information on the Cr_7_Co energy spectrum, by detecting a low-energy transition below 0.1 meV (see the shoulder in Fig. 2). In order to check its magnetic origin, we have extracted the dependence of the INS intensity of this excitation on the two horizontal wavevector components *Q*_*x*_ – *Q*_*y*_, integrated over the full experimental *Q*_*z*_ range (–0.2 to 0.2 Å). The so-obtained experimental intensity map (Fig. 3-a) shows clear **Q**-dependent modulations whose pattern of maxima and minima clearly identify a magnetic transition. The intensity maps have been extracted also for the 0.5 meV peak (Fig. 3-c) and for the 1.8 meV excitation (Fig. S4 of the ESI†). These maps show the same pattern of intensity modulations in the explored **Q** range and all the maxima in each map have the same intensity, demonstrating the same occupation probability for Co^II^ of all the ring sites. Indeed, the differences in the intensity pattern of each excitation are averaged-out by the delocalization of Co^II^ along the ring (intensity maps calculated with the Co ion sitting on one single site are reported in Fig. S5 of the ESI†).

**Fig. 4:**
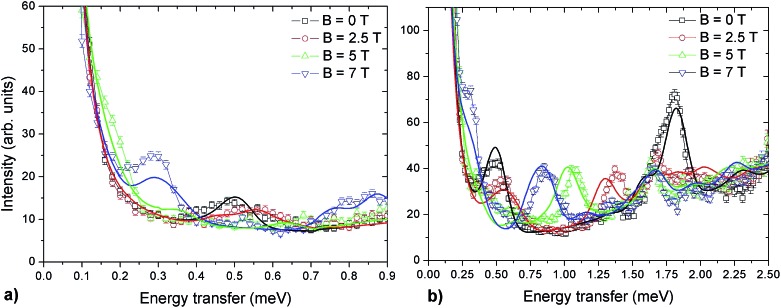
INS spectra for a Cr_7_Co single crystal, collected on the LET spectrometer with an incident neutron energy *E*_i_ = 1.5 meV (a) and *E*_i_ = 3 meV (b) at *T* = 1.8 K in different applied magnetic fields: *B* = 0 T (black squares), *B* = 2.5 T (red circles), *B* = 5 T (green triangles) and *B* = 7 T (blue triangles). Solid lines are simulations based on the spin Hamiltonian in eqn (1) with *J*_Cr–Co_ = 19 K and the local anisotropic exchange tensor obtained from the fitting of all the INS data. The magnetic field is applied in the plane of the ring (see Fig. S6 of the ESI†).

INS experiments on IN5 provide information on the low-lying energy levels of Cr_7_Co up to 6 meV and measurements in temperature allow us to distinguish between transitions involving or not the ground state. However, they are not sufficient to unambiguously identify the Cr–Co exchange parameters. Indeed, IN5 data can be reproduced with a high-anisotropy model (in agreement with DFT + MB results), but also with a weak-anisotropy one. In both cases the antiferromagnetic exchange interactions lead to a *S* = 1 ground multiplet. In the high-anisotropy case both the 0.1 meV and 0.5 meV INS peaks in Fig. 2 are due to intramultiplet transitions within the ground *S* = 1 manifold and therefore a strong and rhombic anisotropic Cr–Co exchange is responsible for the large 0.5 meV splitting of the ground multiplet. In this case the cold transition at 1.8 meV is the lowest-energy intermultiplet transition, yielding a large *J*_Cr–Co_ value, of the same order as *J*_Cr–Cr_. Conversely, if we assume that the 0.5 meV peak is the lowest intermultiplet transition, we have to consider a weak Cr–Co anisotropic exchange leading to a small splitting of the *S* = 1 ground state multiplet of only 0.1 meV. In order to solve this puzzle, we have performed a targeted INS experiment in applied magnetic field. We have measured a single crystal sample of Cr_7_Co on the LET^44^ spectrometer at the ISIS facility, Rutherford Appleton Laboratory (Didcot, UK), at *T* = 1.8 K and with magnetic fields *B* = 0 T, 2.5 T, 5 T and 7 T (experimental details are given in the ESI†). The behaviour of the INS excitations as a function of the applied magnetic field enables us to distinguish the two situations and to evaluate the anisotropy of the molecule. Fig. 4-a shows the high-resolution spectra collected on the LET spectrometer with an incident neutron wavelength *E*_i_ = 1.5 meV and with different applied magnetic fields, while the higher energy-transfer spectra with *E*_i_ = 3 meV are reported in Fig. 4-b. The cold transitions at 0.5 meV and 1.8 meV are clearly visible in the zero-field spectra. A decisive information about the anisotropy of the Cr_7_Co has been provided by the magnetic-field dependence of the position of the lowest-energy cold peak, detected at 0.1 meV at zero-field (see Fig. 2). This transition is not visible in the *B* = 0 T and *B* = 2.5 T LET spectra, as it is underneath the elastic peak. Nevertheless, the peak moves to higher energies by increasing the magnetic field and becomes clearly visible at 0.3 meV with *B* = 7 T (see Fig. 4-a). This lowest-energy INS excitation corresponds to a transition within the *S* = 1 ground multiplet in both the high-anisotropy and weak-anisotropy model. The small observed energy shift (from 0.1 meV to only 0.3 meV) with the application of a strong a magnetic field of 7 T can be explained only by the high-anisotropy model. Indeed, a much larger field-induced shift is expected in the weak-anisotropy one, almost twice as large as the one detected by INS measurements (energy levels as a function of the applied magnetic field calculated with the weak-anisotropy model are reported in Fig. S7 of the ESI†).

The parameters of the spin Hamiltonian in eqn (1) have been determined by fitting all the INS data reported in Fig. 2 and 4, which are very well-reproduced with *J*_Cr–Co_ = 19 ± 2 K and a strong and rhombic Cr–Co anisotropic exchange term with *D*_*zz*_ = –10 ± 1 K and *D*_*yy*_ = 13.0 ± 1 K leading to (in K)3
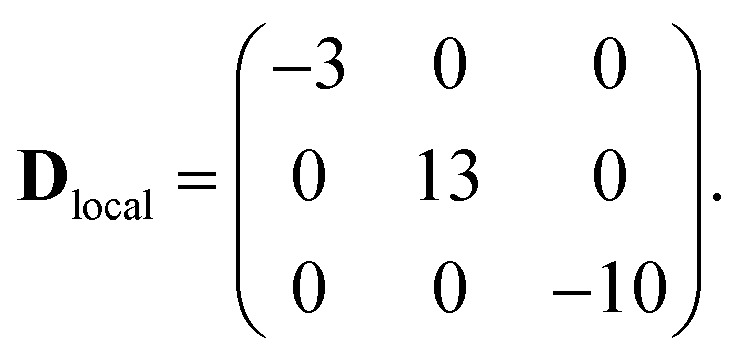



**Fig. 5:**
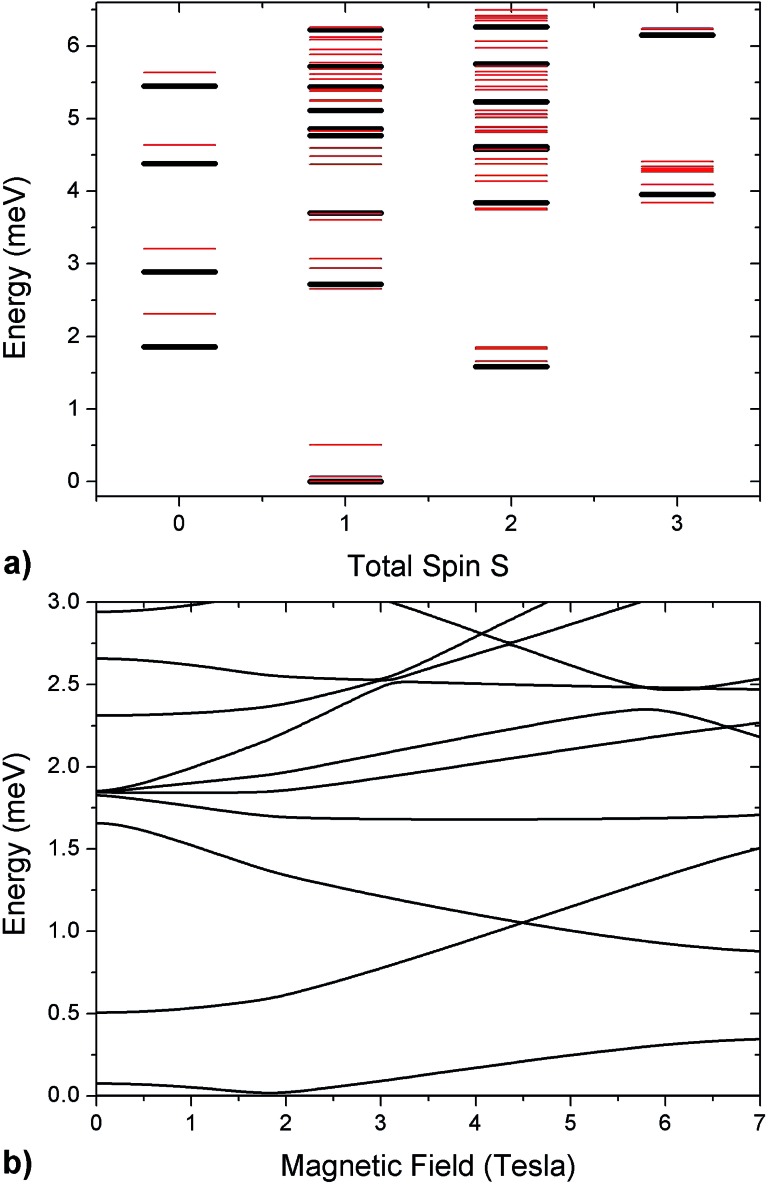
(a) Low-lying energy levels of the Cr_7_Co ring calculated taking into account the isotropic exchange terms of the spin Hamiltonian in eqn (1) with *J*_Cr–Co_ = 19 K (black lines). Red lines are obtained adding the ZFS term and the Cr–Co anisotropic exchange, given the local anisotropic exchange tensor in eqn (3). Since *S* is not a good quantum number for the full spin Hamiltonian, we have assigned to each level the total-spin value *S* with the major components in the corresponding eigenstate. (b) Magnetic field dependence of Cr_7_Co energy levels up to 7 T with the Co^II^ ion on site 8. The magnetic field is applied in the plane of the ring (see experimental configuration in Fig. S6 of the ESI†).

As in the DFT + MB results, there are two directions where the Cr–Co anisotropic exchange is strong but opposite in sign and a third one where it is almost one order of magnitude weaker. The spin Hamiltonian results also yield a strong Cr–Co isotropic exchange interaction and are therefore in good agreement with DFT + MB predictions. It is worth to note that this model also reproduces the IN5 intensity maps in Fig. 3 and S4 of the ESI.† 32,37,43 The calculated low-lying energy levels and their magnetic field dependence are reported in Fig. 5.

## 3 Discussion

The analysis of the EPR and INS experimental data supported by our DFT + MB calculations have allowed us to characterize the anisotropy of the Cr_7_Co ring, which has proven to be an ideal test system to study anisotropic exchange interactions. Our results demonstrate that the insertion of an anisotropic Co ion strongly coupled to the Cr ones determines the anisotropy of the whole molecule. Indeed, the strong and rhombic Cr–Co anisotropic exchange leads to a splitting of the *S* = 1 ground multiplet *Δ* = 0.5 meV, which is five times larger than the splitting of the *S* = 1 ground manifold of the parent compound Cr_7_Mn.^33^ Since Mn^II^ is essentially isotropic in Cr_7_Mn, the comparison between these two experimental splittings is practically a direct comparison between the effects of Cr–Co anisotropic exchange and of the seven Cr single-ion anisotropies. The ZFS due to the Cr ions is very similar in both Cr_7_Mn and Cr_7_Co and leads to a very small splitting of the *S* = 1 ground state. Indeed, if we neglect the Cr–Co anisotropic exchange, we obtain a splitting of about 0.1 meV in Cr_7_Co, practically identical to Cr_7_Mn. Thus, by inserting only one anisotropic ion we obtain a five times larger splitting of the ground state, confirming that the experimentally observed splitting is mostly due to the anisotropic exchange between Co^II^ and the neighbouring Cr ions. The anisotropy of Cr_7_Co is also responsible for a strong *S*-mixing^41^ in the eigenstates of the molecule and therefore the total-spin *S* is not a really good quantum number for the full spin Hamiltonian in eqn (1). For instance, the strong Cr–Co anisotropic exchange leads to a mixing between the *S* = 1 ground multiplet and excited multiplets of about 10% (mainly with the lowest *S* = 2 manifold). This strong *S*-mixing significantly affects the physics of Cr_7_Co leading, for instance, to oscillations of the total spin^45^,^46^ for specific field values. Large anticrossings associated with these oscillations are induced by a magnetic field applied perpendicular to the plane of the ring (see Fig. S8 of the ESI†). In addition, *S*-mixing can play an important role in the dynamics of the Néel vector.^28^

It is worth noting that the effective anisotropic exchange interaction in eqn (1) originates from the combined effect of the Co^II^ ion zero-field splitting and of a real Cr–Co anisotropic exchange. In fact, our DFT + MB results show that the ***g***_Co_ tensor, which reflects the single-ion anisotropy of the Co^II^, and the **D**_local_ tensor, accounting for the effective Cr–Co anisotropic exchange, have different local principal axis (see Fig. 1). In order to better understand the effects of the Cr–Co anisotropic exchange on the magnetic behaviour of the molecule at low temperatures, we have applied second-order perturbation theory to project the Cr–Co anisotropic exchange interactions onto an effective *S* = 1 multiplet.^41^ With this procedure we have determined the principal axis of the effective anisotropy tensor acting on the *S* = 1. The easy-axis of the effective anisotropy lies within the plane of the ring and points in the radial direction from the centre of the ring towards the Co ion, whereas one of the two hard directions is perpendicular to the plane of the ring.

This study has allowed us to understand the nature of the Cr–Co anisotropic exchange interactions in the Cr_7_Co ring and their effects on the magnetic behaviour of the molecule. Moreover, it also represents an important starting point for the design of new systems where strong exchange interactions transmit the large anisotropy of Co^II^ ions to the whole molecule. As a first attempt, more than one Co ion can be embedded in the antiferromagnetic ring to further increase the anisotropy of the cluster.^47^ Having fully characterized the Cr–Co anisotropic exchange, we are able to design the molecule in order to maximize the anisotropy-induced splitting of the ground states (which will be an *S* = 2 with two Co^II^ ions within the ring). For instance, Co ions on opposite sites (*e.g.* sites 1 and 5 in Fig. 1) will produce a splitting about 40% larger than that obtained when the two Co ions have one Cr^III^ in between (*e.g.* sites 6 and 8 in Fig. 1).§
§Exchange of a protonated amine for an imidazolium cation enables the introduction of a second divalent ion in AF rings,^48^ although this does result in multiple isomers due to disorder of the divalent ion about the eight metal sites. However, this family of compounds exhibit considerable synthetic flexibility from which many derivatives are possible.^49^,^50^ Thus, opposing divalent sites can be obtained by changing the structure of the cycle.^51^


The inclusion of anisotropic ions strongly coupled to high-spin ones (like Mn^II^, Fe^III^ or Cr^III^) in polymetallic clusters is important also in view of exploiting MNMs for quantum information processing (QIP). In particular, a crucial milestone would be to reach the so-called strong coupling between a single magnetic molecule and the quantized magnetic field (photons) of coplanar superconducting resonators.^38^ Indeed, this would allow the local control and read out of the molecule magnetic state^38^,^52^ and to implement QIP schemes similar to those used for superconducting qubits.^53^,^54^ The large spin-photon coupling needed to reach the strong coupling regime could be achieved with a molecule characterized by a large total spin and by a strong easy-plane anisotropy.^38^ These are often conflicting requirements, but the present results demonstrate that the inclusion of one or more anisotropic 3d ions in a high-spin polymetallic complex is a promising way of achieving this very important goal. At last, anisotropic ions coupled with molecular qubits can be used as a switch of the effective qubit–qubit interaction in the implementation of quantum gates.^26^

## 4 Conclusions

We have studied the Cr_7_Co ring as a model system to understand how the insertion of an anisotropic Co ion strongly coupled to the Cr ones affects the anisotropy of the whole molecule. Since the ring is isostructural with the other previously studied Cr_7_M AF rings, we have been able to focus on Co^II^ single-ion anisotropy and on Cr–Co anisotropic exchange interactions. This characterization has been possible thanks to a combined use of EPR and INS techniques with spin Hamiltonian and DFT + MB calculations. EPR measurements on Ga_7_Co, where Ga^III^ is diamagnetic, have allowed us to determine the strong single-ion anisotropy of Co^II^ embedded in the ring. Then, the Cr_7_Co energy spectrum has been surveyed with a broad set of INS experiments. In-field INS measurements have been crucial to determine the Cr–Co anisotropic exchange parameters and therefore the anisotropy of the molecule. DFT + MB results have been used as a guide for fitting the spin Hamiltonian parameters and their agreement with experiments is good, especially considering the presence of an highly-anisotropic ion like Co and the many anisotropic terms in the spin Hamiltonian.

We have found strong anisotropic exchange interactions between Co^II^ and the neighbouring Cr ions. Thus, our results demonstrate that the anisotropy of Co^II^ is efficiently transmitted to the anisotropy of the whole Cr_7_Co polymetallic cluster through strong effective anisotropic exchange interactions.

This study is also a starting point for the design of new systems, where strong exchange interactions transmit the large anisotropy of Co^II^ ions to the whole molecule. On the one hand, a rich physics is expected in these systems, due to unquenched orbital degrees of freedom (*e.g.* Néel Vector Tunneling in the low-frequency dynamics). On the other hand, the combination of high-spin ions strongly coupled to a few very-anisotropic ions like Co^II^ represents a promising route for building scalable quantum information architectures.

## Conflicts of interest

There are no conflicts to declare.

## Supplementary Material

Supplementary information

Crystal structure data
