# Correction: Anisotropy of Co^II^ transferred to the Cr_7_Co polymetallic cluster *via* strong exchange interactions

**DOI:** 10.1039/c8sc90065e

**Published:** 2018-03-23

**Authors:** Elena Garlatti, Tatiana Guidi, Alessandro Chiesa, Simon Ansbro, Michael L. Baker, Jacques Ollivier, Hannu Mutka, Grigore A. Timco, Inigo Vitorica-Yrezabal, Eva Pavarini, Paolo Santini, Giuseppe Amoretti, Richard E. P. Winpenny, Stefano Carretta

**Affiliations:** a Dipartimento di Scienze Matematiche, Fisiche e Informatiche , Università di Parma , I-43124 Parma , Italy . Email: stefano.carretta@unipr.it; b ISIS Facility , Rutherford Appleton Laboratory , OX11 0QX Didcot , UK; c Institute for Advanced Simulation , Forschungszentrum Jülich , 52425 Jülich , Germany; d The School of Chemistry , Photon Science Institute , The University of Manchester , M13 9PL Manchester , UK; e Institut Laue-Langevin , 71 Avenue des Martyrs CS 20156 , Grenoble Cedex 9 F-38042 , France; f The School of Chemistry , The University of Manchester at Harwell , Didcot , OX11 0FA , UK; g JARA High-Performance Computing , RWTH Aachen University , 52062 Aachen , Germany

## Abstract

Correction for ‘Anisotropy of Co^II^ transferred to the Cr_7_Co polymetallic cluster *via* strong exchange interactions’ by Elena Garlatti *et al.*, *Chem. Sci.*, 2018, DOI: ; 10.1039/c8sc00163d.



## 

In the published article eqn (1) contained misprints. The correct form of the eqn is shown below:1
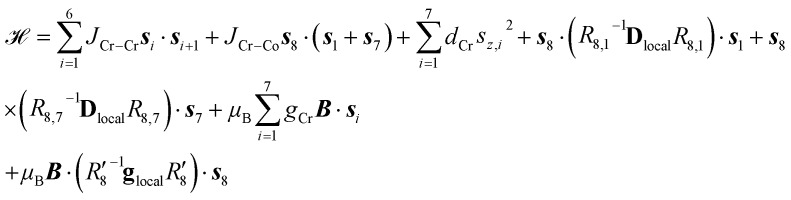



The Royal Society of Chemistry apologises for these errors and any consequent inconvenience to authors and readers.

