# Supplementary material for: Anisotropy of CoII transferred to the Cr7Co polymetallic cluster via strong exchange interactions
Source: Chem Sci. 2018 Mar 7;9(14):3555–62. doi: 10.1039/c8sc00163d (PMC5934825; doi:10.1039/c8sc00163d)
Supplement: Supplementary file 1 [file SC-009-C8SC00163D-s001.pdf]

Electronic Supplementary Material (ESI) for Chemical Science.
This journal is © The Royal Society of Chemistry 2018

Supplementary information

Anisotropy of Co^{II} transferred to the Cr_7Co polymetallic cluster via strong exchange interactions

Elena Garlatti, Tatiana Guidi, Alessandro Chiesa, Simon Ansbro, Michael L. Baker, Jacques Ollivier, Hannu Mutka, Grigore A. Timco, Inigo Vitorica-Yrezabal, Eva Pavarini, Paolo Santini, Giuseppe Amoretti, Richard E. P. Winpenny and Stefano Carretta

Synthesis

$[Co_2(O_2CCMe_3)_4(HO_2CCMe_3)_4(H_2O)]$ was obtained by a procedure given in [1]. All the other reagents and solvents were commercially available and used as received.
$\{[NH_2Me_2][Cr_7CoF_8(O_2CCMe_3)_{16}]\}$ was prepared based on the method given in [2], but with small modifications.
Chromium(III) fluoride tetrahydrate (5.0 g, 27.6 mmol) and $[Co_2(O_2CCMe_3)_4(HO_2CCMe_3)_4(H_2O)]$ (2.5 g , 2.63 mmol), dimethylammonium dimethylcarbamate (0.7 g, 5.22 mmol) and pivalic acid (30.0 g, 294 mmol) where heated while stirring at 160° C for 26 h. The solution was allowed to cool to room temperature and acetone added to cause complete precipitation of the target compound. The crystalline product was collected by filteration, washed with a large quantity of acetone and recrystallized from pentane and dried in air. Yield 8.3g (94%).
X-Ray quality crystals of $\{[NH_2Me_2][Cr_7CoF_8(O_2CCMe_3)_{16}]\} \cdot Et_2O$, including large single crystals suitable for use for the INS measurements were obtained by crystallization of the Cr_7Co ring from a mixture of Et_2O/MeCN, by slow evaporation of the solvents at ambient temperature.
Elemental analysis, calcd (%) for $C_{86}H_{162}CoCr_7F_8NO_{33}$: Cr 15.74, Co 2.55, C 44.66, H 7.06, N 0.61; Found: Cr 15.67, Co 2.54, C 44.95, H 7.07, N 0.64.

Single Crystal X-ray Diffraction

Data Collection. X-ray data for compound $\mathbf{[(CH_3)_2NH_2][Cr_7CoF_8(O_2CC(CH_3)_3)_{16}]\cdot(CH_3CH_2O)}$ was collected at a temperature of 150 K using a using Mo-K$_\alpha$ radiation on a RIgaku_Oxford supernova diffractometer, equipped with Eos detector and an Oxford Cryosystems Cobra nitrogen flow gas system. Data were measured using CrysAlisPro suite of programs.
Crystal structure determinations and refinements. X-ray data were processed and reduced using CrysAlisPro suite of programs. Absorption correction was performed using empirical methods based Empirical absorption correction was performed using spherical harmonics, implemented in the SCALE3 ABSPACK scaling algorithm [3, 4]. Crystal structures were solved and refined against all F^2 values using the SHELX and Olex 2 suite of programs [5, 6, 7]. All the atoms were refined anisotropically. Hydrogen atoms were placed in calculated positions, refined using idealized geometries (riding model) and assigned fixed

isotropic displacement parameters. Hydrogens corresponding to the methyl groups of the dimethylamonium group were omitted in the model but included in the formula. The Cr and Co atoms were refined with fixed occupancies 7/8 and 1/7 respectively. The atomic displacement parameters and positions were constrained to be equal using SHELX EADP and EXYZ commands.

CCDC **1810773** contains the supplementary crystallographic data for this paper. These data can be obtained free of charge *via* www.ccdc.cam.ac.uk/conts/retrieving.html (or from the Cambridge Crystallographic Data Centre, 12 Union Road, Cambridge CB21EZ, UK; fax: (+44)1223-336-033; or deposit@ccdc.cam.ac.uk).

Table S1. Crystallographic information for compound **$[(CH_3)_2NH_2][Cr_7CoF_8(O_2CC(CH_3)_3)_{16}]\cdot(CH_3CH_2O)$**

	**$[(CH_3)_2NH_2][Cr_7CoF_8(O_2CC(CH_3)_3)_{16}]\cdot(CH_3CH_2O)$**
Crystal colour	Green
Crystal size (mm)	0.40 × 0.40 × 0.20
Crystal system	Tetragonal
Space group, *Z*	I4, *2*
a (Å)	19.9066(6)
c (Å)	16.1991(9)
V ($Å^3$)	6419.3(5)
Density ($Mg.m^{-3}$)	1.197
Wavelength (Å)	0.71073
Temperature (K)	150
μ(Mo-Kα) (mm^{-1})	0.768
2Θ range (°)	6.636 to 58.51
Reflns collected	93408
Independent reflns (R_{int})	15604 (0. 0683)
L.S. parameters, *p*	325
No. of restraints, *r*	282
$R1$ $(F)^a$ $I > 2.0\sigma(I)$	0.0675
$wR2(F^2)$,[b] all data	0.2016
$S(F^2)$,[c] all data	1.051

[a] $R1(F) = \Sigma(|F_O| - |F_C|)/\Sigma|F_O|$; [b] $wR2(F^2) = [\Sigma\, w(F_O^2 - F_C^2)^2/\Sigma\, wF_O^4]^{½}$; [c] $S(F^2) = [\Sigma\, w(F_O^2 - F_C^2)^2/(n + r - p)]^{½}$

DFT+MB calculations

In order to derive the effective low-energy spin Hamiltonian of the examined system, we adopt the DFT+MB technique described in [8] and [9]. We initially perform DFT calculations in the local density approximation (LDA). Since the molecule studied in the present work have a large number of atoms, these calculations can be rather time-consuming. To perform them we employ the NWCHEM code [10], which is optimized to exploit the power of modern massively parallel supercomputers, and a triple-ζ valence basis set of Gaussians. Next we identify and select the transition metal d -like states around the Fermi level. By using the Foster-Boys localization procedure [11] we then build a set of localized orbitals centred on the transition metal ions, which span such low-energy states. From this set of orbitals we calculate the crystal-field Foster-Boys states by diagonalizing the on-site part of the one-electron Hamiltonian (nonrelativistic calculation). We thus obtain system-specific Hubbard models (in second quantization form):

$$H = -\sum_{ii'} \sum_{mm'\sigma} t^{ii'}_{mm'} c^{\dagger}_{im\sigma} c_{i'm'\sigma} + \frac{1}{2} \sum_{ii'} \sum_{\sigma\sigma'} \sum_{\substack{mm' \\ pp'}} U^{ii'}_{mpm'p'} c^{\dagger}_{im\sigma} c^{\dagger}_{i'p\sigma'} c_{ip'\sigma'} c_{i'm'\sigma}$$
$$+ \sum_{i} \lambda_i \sum_{mm'} \sum_{\sigma\sigma'} \xi^{i}_{m\sigma,m'\sigma'} c^{\dagger}_{im\sigma} c_{im'\sigma'} - H_{DC}$$

Here $c^{\dagger}_{im\sigma}$ ($c_{im\sigma}$) creates (annihilates) a 3d electron with spin σ in the crystal-field Boys orbital m at site i.

The parameters $t^{ii'}_{mm'}$ are the hopping integrals ($i \neq i'$) or the crystal-field matrix ($i = i'$), while $U^{ii'}_{mpm'p'}$ are the screened Coulomb integrals. The term H_{DC} is the double counting correction, which removes the part of the Coulomb interaction already included and well accounted for in the LDA; λ_i is the spin-orbit coupling. The results presented in this work are obtained neglecting terms with more than two orbital indices and using the rotational invariant form of the Coulomb vertex, including spin-flip and pair hopping terms but (for simplicity) no Coulomb anisotropy; thus all Coulomb parameters can be expressed as a function of the averaged screened Coulomb couplings U^{ii} and J^{ii} which, in turn, depend only on the Slater integrals F_0, F_2, and F_4 [12].

The essential Coulomb terms are thus the direct [$U^{ii}_{mm'mm'} = U^{ii} - 2J^{ii}(1 - \delta_{mm'})$] and the exchange [$U^{ii}_{mm'm'm} = J^{ii}$] interaction, the pair-hopping term [$U^{ii}_{mmm'm'} = J^{ii}$] and the spin-flip term [$U^{ii}_{mm'm'm} = J^{ii}$]. The last two interactions are crucial to determine the correct structure of the spin multiplets. We determine the latter by using the constrained local density approximation [13] approach in the Foster-Boys basis, keeping the basis frozen in the self-consistency loop. The term H_{DC} is the double-counting correction, which removes the mean-field part of the local Coulomb interaction, already included in the LDA. Here we adopt the fully localized limit [12], which is more appropriate for molecular systems. Hence $H_{DC} = \frac{1}{2}\sum_i U^{ii} n^i_d \left(n^i_d - 1\right) - \frac{1}{2}\sum_i J^{ii} n^i_d \left(\frac{1}{2} n^i_d - 1\right)$, where n^i_d is the number of d electrons at site i. Finally, λ_i is the strength of the spin-orbit interaction, here the same for all the 3d electrons within the same ion. The elements $\xi^{i}_{m\sigma,m'\sigma'} = \langle m\sigma | \mathbf{s}^i \cdot \mathbf{l}^i | m'\sigma' \rangle$ are matrix elements of the spin-orbit matrix in the Forster-Boys basis. The coupling λ_i can be extracted by comparing the single-electron crystal-field splittings with and without spin-orbit interaction, as we have shown in Ref. [8]. In this work, since relativistic self-consistent calculations are very time-consuming and the spin-orbit coupling is basically a property of the single ion (in its local environment, which for all systems considered here is approximately octahedral), for λ_i we use for Cr^{III} values previously determined by us via our method in the same crystal field environment [8] and for

Co[II] tabulated values for the free ion [14], with the same screening factor obtained in a similar environment. We had indeed proved in Ref. [1] that the latter is a very good approximation for this kind of systems. For the Co[II] ion we get $U^{ii} = 6.5$ eV, $J^{ii} = 0.45$ eV and $\lambda_i = 33$ meV.

In order to gain a deeper insight into the behaviour of Co(II) in its particular coordination environment, we report in Figure S1 its calculated lowest lying Kramers doublets.

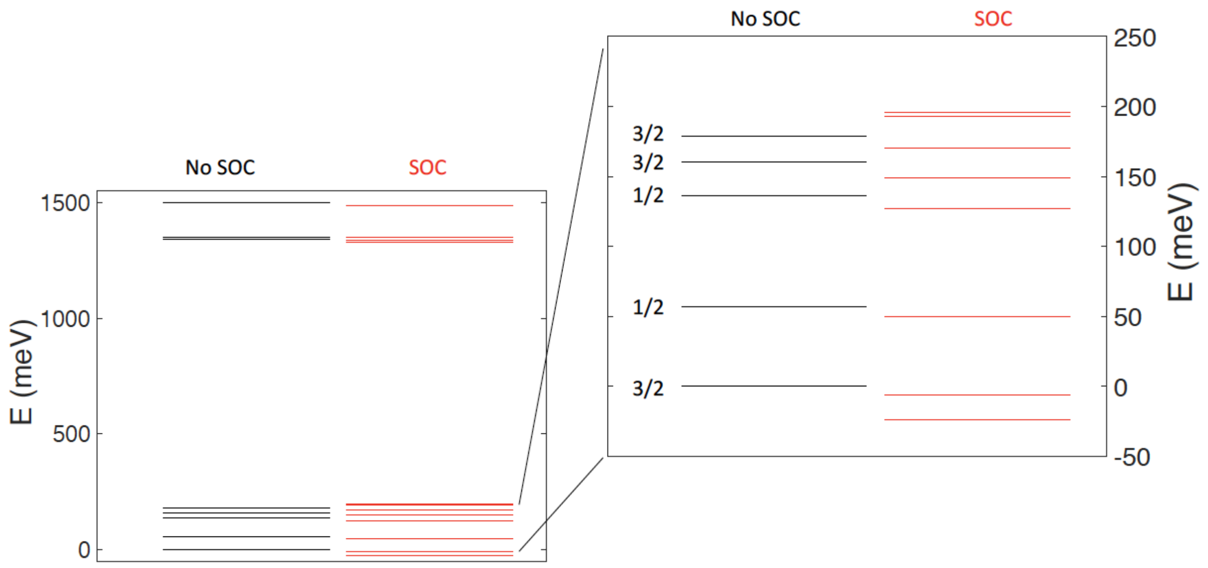

Figure S1 Level diagram of Co(II) ion computed by DFT+MB approach, with (red lines) and without (black) spin-orbit interaction.

These arise from the combined action of crystal field splitting, Coulomb and spin-orbit interaction. In the Figure, we separate these two effects by comparing the level diagrams with (red lines) and without (black) the inclusion of spin orbit coupling (SOC). In the zoom on the lowest-lying levels reported in the right panel the total spin of each multiplet (without SOC) is also indicated.

The highly anisotropic behavior of Co(II) results from the presence of low-lying excited states (the first excited doublet being at 180 K). This yields a large anisotropy, as can be understood by using second-order perturbation theory to compute the exchange matrix in the low-energy subspace spanned by the lowest multiplets of ions 1, 8. This is given by:

$$\langle \psi_k^1(n_1)\psi_j^8(n_8)|H_{ex}|\psi_{k'}^1(n_1)\psi_{j'}^8(n_8)\rangle$$
$$= \sum_{l,m} \frac{\langle \psi_k^1(n_1)\psi_j^8(n_8)|H_t|\psi_l^1(n_1 \pm 1)\psi_m^8(n_8 \mp 1)\rangle\langle \psi_l^1(n_1 \pm 1)\psi_m^8(n_8 \mp 1)|H_t|\psi_{k'}^1(n_1)\psi_{j'}^8(n_8)\rangle}{E_k^1(n_1) + E_j^8(n_8) - E_l^1(n_1 \pm 1) - E_m^8(n_8 \mp 1)}$$

where $H_t = -\sum_{mm'\sigma} t_{mm'}^{18} c_{1m\sigma}^{\dagger} c_{8m'\sigma} + \mathrm{h.c.}$ is the hopping term of the Hubbard model (reported in the ESI) between sites 1 and 8, $\psi_k^i(n_i)$ are single-ion eigenstates on ion *i* with n_i electrons and energy $E_k^i(n_i)$. In the above equation, k, k', j, j' run over the ground multiplets of the two examined ions (the Co[II] doublet and the Cr[III] quartet) and l, m over the excited ones.

Since the single ion eigenstates already include the effect of SOC, anisotropic exchange interactions in the above expression arise from the product of matrix elements of the SOC and inter-site hoppings [15]. The directional character of the latter, combined with the Co(II) single-ion anisotropy leads to the $\boldsymbol{D}_{local}$ reported in the text.

In the table below we report the director cosine of $\mathbf{g}_{local}$ and $\mathbf{D}_{local}$

	X	Y	Z
g_{local}^{x}	0.70	0.69	0.15
g_{local}^{y}	-0.71	0.70	0.08
g_{local}^{z}	-0.06	0.16	0.98
D_{local}^{x}	-0.07	0.05	-0.99
D_{local}^{y}	-0.38	-0.92	0.08
D_{local}^{z}	-0.92	0.38	0.03

Spin Hamiltonian calculations

In order to evaluate the energy spectrum of Cr_7Co, we have exploited the irreducible tensor operators (ITO's) technique. The spin Hamiltonian H in Eq. 1 has been rewritten in terms of ITO's and their matrix elements on a total-spin basis have been calculated by exploiting the Wigner-Eckart theorem and the recoupling technique. For the calculation of the matrix elements, the diagonalization of the Hamiltonian matrix and the simulation of experimental data a specific Fortan90 code has been written.
The dimension of the Hamiltonian matrix increases dramatically with the number of magnetic centres and with the local spin values. In order to diagonalize the Hamiltonian in Eq.1, we have therefore followed a two-step procedure [16]. Since the Heisenberg interaction represents the dominant contribution, in the first step of the diagonalization procedure only the first term in Eq. 1 has been considered. By exploiting the rotational invariance of the isotropic exchange term, the Heisenberg Hamiltonian matrix has been block-factorized according to the total spin quantum number S and the corresponding eigenvalues and eigenvectors have been calculated. However, the anisotropic terms of H do not commute with the total squared spin operator S^2 and mix different total-spin subspaces (S mixing) [17] and in the particular case of Cr_7Co, the strong anisotropy terms lead to a very strong S-mixing. In order to reduce the computational effort, we have retained only the exchange eigenvectors with eigenvalues lower than a threshold. Within this reduced subspace, all magnetic interactions can be evaluated including also the often-neglected S mixing effects, and the energy spectrum of the complete Hamiltonian in Eq. 1 can be obtained. Calculations show that the reduction to the subspace spanned by the lowest exchange manifolds (up to E/k = 120 K) allows us to reproduce properly the low-temperature properties, reducing the computational effort.

EPR spectroscopy on Ga_7Co

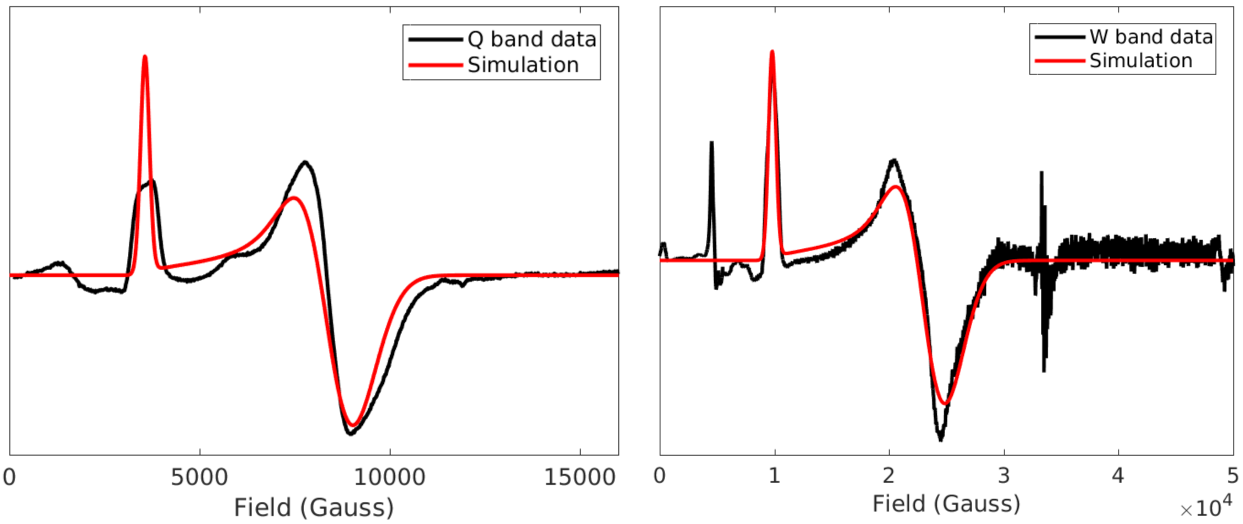

Figure S2: Left: Q-band EPR spectrum measured at 5 K (black line). Simulation (red line). Right: W-band EPR spectrum of Ga7Co measured at 5 K (black line). Simulation (red line).

Q-band (34 GHz) and W-band (94 GHz) EPR spectra of polycrystalline powders were recorded at W-band on a Bruker Elexsys E600 and at Q-band on a Bruker EMX spectrometers. The data were collected at a sample temperature of 5.0 K. The measured spectra are baseline subtracted using a polynomial baseline fitting procedure. EPR simulations include g-strain type FWHM broadening of 0.5 for the g principle values.

INS spectroscopy

IN5 measurements on powders

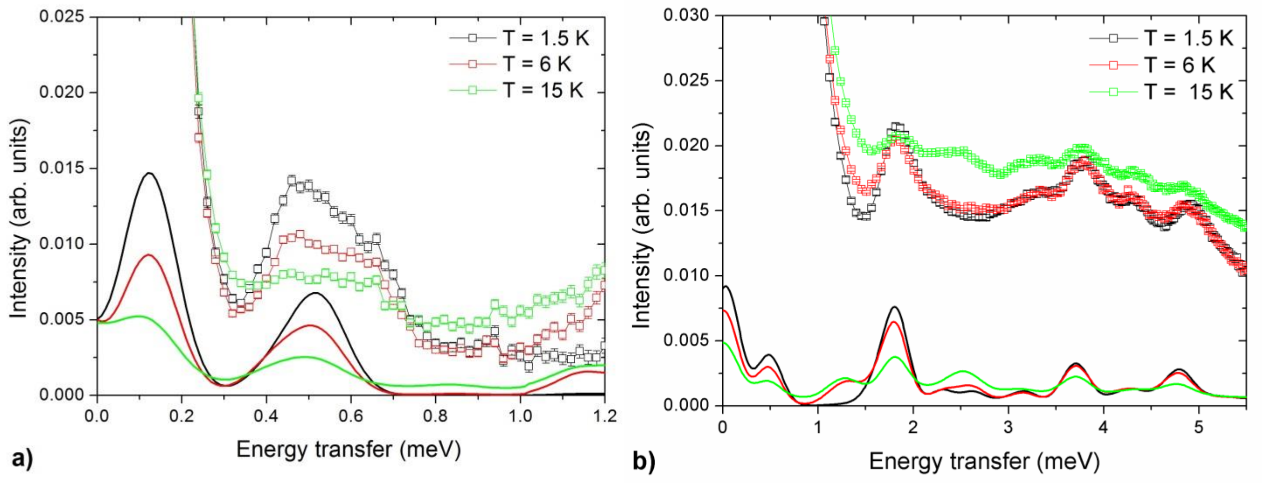

Figure S3: INS data for the Cr_7Co ring measured on powder samples on th IN5 spectrometer. a) High-resolution/low-energy-transfer spectra measured with an incident neutron wavelength λ = 6.5 Å at 1.5 K (black squares), 6 K (red squares), and 15 K (green squares). b) High-energy-transfer spectra measured with an incident neutron wavelength λ = 3.2 Å at 1.5 K (black squares), 6 K (red squares), and 15 K (green squares). The solid lines show simulations based on the spin Hamiltonian in Eq.1 and with J_{Cr-Co} = 19 K and the local anisotropic exchange tensor in Eq. 3.

Inelastic neutron scattering was performed on the IN5 time of flight inelastic spectrometer at Institute Laue-Langevin, Grenoble, France. A polycrystalline non-deuterated sample of Cr7Co was loaded into a

hollow aluminum cylinder for measurement. An ILL orange vertical cryomagnet was used to maintain sample temperatures down to 1.5 K. The measurements were performed in zero applied magnetic field. The INS energy spectra include scattering intensity from all detector angles. Detector efficiencies were normalized to a standard vanadium measurement, and were background corrected via the subtraction of sample environment contributions. Measurements were performed with incident neutron wavelengths of 6.5 and 3.2 Å, using chopper speeds of 9600 and 8000 revolutions per minute respectively, providing energy resolutions at zero energy transfer with full width half maxima of 45 μeV and 0.4 meV respectively.

IN5 measurements on single crystals

We have investigated a single crystal sample of the antiferromagnetic ring Cr_7Co on the time-.of-flight spectrometer IN5. By utilizing its position sensitive detectors we have determined the S(Q,ω) function over a large portion of reciprocal space and in the desired energy transfer range. The crystal was mounted with the *c* axis perpendicular to the horizontal scattering plane on a specially made aluminum sample holder and placed inside an annular aluminum can, with some excess solvent and sealed under helium. Spectra were collected at 3.2 and 6.5 Å, with an instrumental energy resolution of 350 and 90 μeV FWHM at zero energy transfer respectively, at a temperature of 1.5K. The Q-dependence of the excitations were surveyed by rotating the sample throughout the full 360 degrees and measuring at every degree.

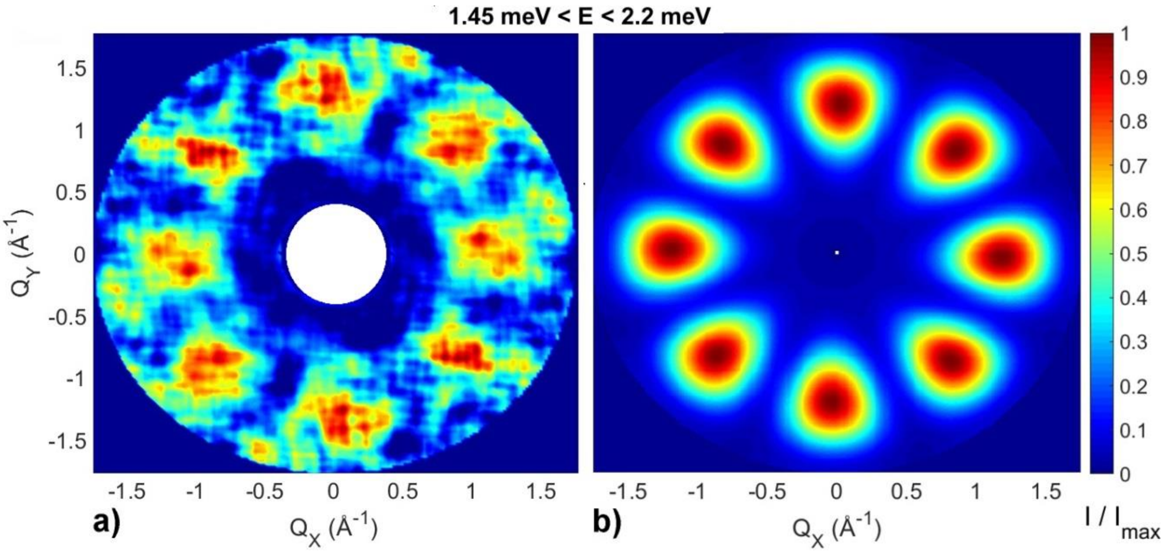

Figure S4: Constant-energy plots of the neutron scattering intensity measured on IN5 with a 3.2 Å incident neutron wavelength and with a sample temperature of 1.5 K. Panel **a** show the experimental dependency of the neutron scattering intensity of the inelastic excitations observed at 1.8 meV on the two horizontal wavevector components Q_x - Q_y, integrated over the full experimental Qz data range (-0.2 - 0.2 Å). The measured cross-section has been integrated over an energy range centred around the observed transition energy: 1.45 – 2.2 meV. The colour bar reports the transition intensity normalized for the maximum in each panel. Panel **b** report the corresponding calculations based on the spin Hamiltonian in Eq.1 with J_{Cr-Co} = 19 K and the local anisotropic exchange tensor in Eq. 3, taking into account the delocalization of the Co ion along the ring.

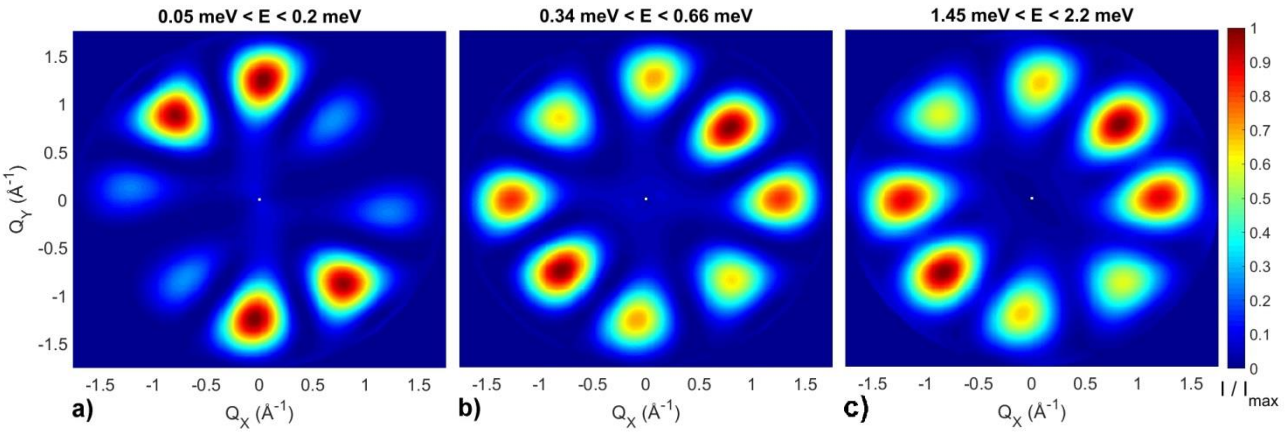

Figure S5: Constant-energy plots of the neutron scattering intensity calculated with the Co ion on site 8. They reproduce the dependency of the neutron scattering intensity of the inelastic excitations observed at 0.1 meV (a), 0.5 meV (b) and 1.8 meV (c) on the two horizontal wavevector components Q_x - Q_y, integrated over the full experimental Qz data range (-0.2 - 0.2 Å) and over an energy range centred around the observed transition. The colour bar reports the transition intensity normalized for the maximum in each panel. These calculations are based on the spin Hamiltonian in Eq.1 with J_{Cr-Co} = 19 K and the local anisotropic exchange tensor in Eq. 3.

Spectra as a function on energy transfer in Figure 2 have been obtained integrating over the full Q_x-Q_y-Q_z range. The full experimental intensity maps in Figure 3 and S4 has been reconstructed using the HORACE analysis suite [18]. In order to isolate the magnetic signal from the non-magnetic response, we integrate the data over the full Q_z range and fit, for each set (Q_x,Q_y), the resulting energy dependence with a gaussian plus a background contribution. Corrections for attenuation as a function of rotation and scattering angle were performed by using the intensity modulations of the background signal.

LET measurements in applied magnetic field

The LET experiment was performed on a non-deuterated Cr7Co single crystal of approximate dimensions 10x6x3 mm. The crystal was mounted with the (H H L) plane in the horizontal scattering plane and sealed under helium in an Aluminum can with some excess solvent. The sample was then inserted in a 9 Tesla cryomagnet and cooled down to a base temperature of 1.8 K. The measurements were performed with multiple incident energies E_i = 1.5, 3 and 8.5 meV (90 µeV , 160 µeV and 350 µeV FWHM energy resolution at the elastic line, respectively) and by rotating the crystal by 20 degrees (2 degrees step) about the vertical crystallographic axis [1 -1 0]. Data were collected with the magnetic field applied along the [1 -1 0] direction and with fields of B = 0 T, 2.5 T, 5 T and 7 T. The data shown in Fig. 4 in the main text have been integrated over the full available Q-range, after masking out the spurious signal originating from the scattering from the tail of the cryomagnet.

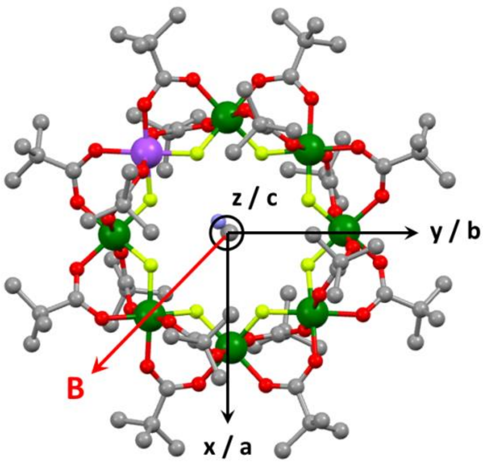

Figure S6: Direction of the applied magnetic field (1,-1,0) for the INS experiment on LET, with respect to the laboratory/unit cell reference system (Co in purple, on site 8).

Energy levels

The weak-anisotropy model

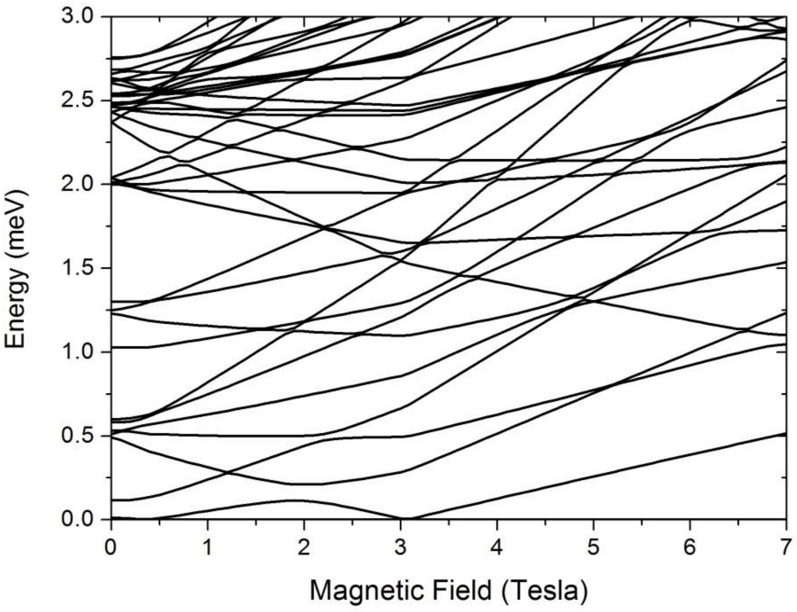

Figure S7: Magnetic field dependence of Cr_7Co energy levels up to 7 T with the Co^{II} ion on site 8, calculated with the spin Hamiltonian in Eq. 1 with $J_{Cr\text{-}Co}$ = 2.1 K and D = -0.4 K. The magnetic field is applied in the plane of the ring (see Figure S6).

Magnetic field perpendicular to the plane of the ring

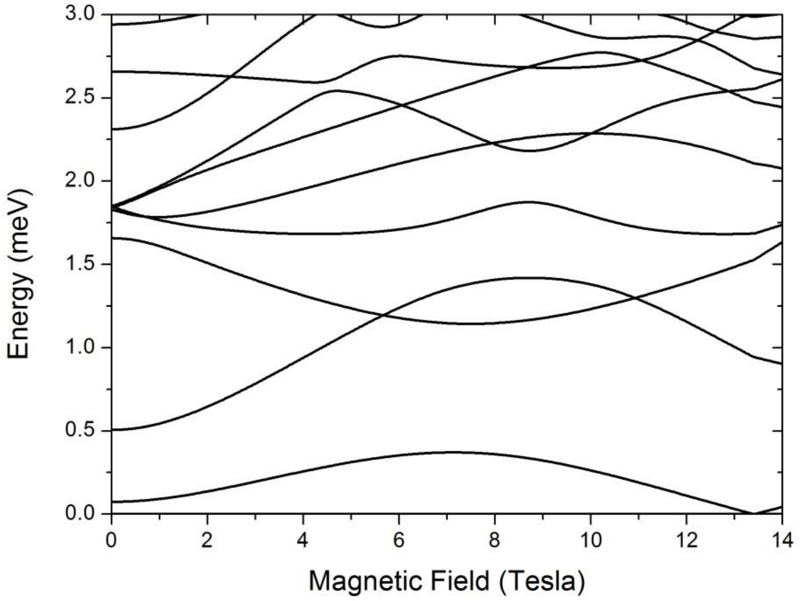

Figure S8: Magnetic field dependence of Cr_7Co energy levels up to 14 T, calculated with the spin Hamiltonian in Eq. 1 with $J_{Cr\text{-}Co}$ = 19 K and the local anisotropic exchange tensor in Eq. 4. Since the magnetic field is perpendicular to the plane of the ring the energy levels as a function of the field intensity don't depend on the position of the Co ion along the ring.

References

1. Aromí G., Batsanov A. S., Christian P., Helliwell M., Parkin A., Parsons S., Smith A. A., Timco G.A., Winpenny R.E.P., *Chem. Eur. Journal*, **2003**, 9, 5142-5161.
2. Larsen K., McInnes E. J. L., El Mkami H., Overgaard J., Piligkos S., Rajaraman G., Rentschler E., Smith A. A., Smith G. M., Boote V., Jennings M., Timco G. A., Winpenny R. E. P., *Angew. Chem. Int. Ed*., **2003**, 42, 101-105.
3. Sheldrick, G. M., SADABS, University of Göttingen, 1995, Empirical absorption correction program based upon the method of Blessing.
4. Blessing, R. H., *Acta Crystallogr*. **1995**, *A51*, 33.
5. Sheldrick, G. M., *Acta Crystallogr*. **2008**, *A64*, 112
6. Sheldrick, G. M., *Acta Crystallogr.* **2015**, *A71*, 3.
7. Dolomanov O.V., Bourhis, L. J., Gildea, R. J., Howard, J. A. K., Puschmann, H., *J. Appl. Cryst.,* **2009**, *42*, 339–341.
8. Chiesa, A.; Carretta, S.; Santini, P.; Amoretti, G.; Pavarini, E. Phys. Rev. Lett. **2013**, 110, 157204.
9. Chiesa, A.; Carretta, S.; Santini, P.; Amoretti, G.; Pavarini, E. Phys. Rev. B **2016**, 94, 224422.
10. Valiev, M.; Bylaska, E. J.; Govind, N.; Kowalski, K.; Straatsma, T. P.; van Dam, H. J. J.; Wang, D.; Nieplocha, J.; Apra, E.; Windus, T. L.; de Jong, W. A. *Comput. Phys. Commun.* **2010**, *181*, 1477.
11. Boys, S. F.; *Rev. Mod. Phys.* **1960**, *32*, 296.
12. *The LDA+DMFT Approach to Strongly Correlated Materials*, edited by Pavarini, E.; Koch, E.; Lichtenstein, A.; Vollhardt, D. (Verlag des Forschungszentrum Jülich, 2011).
13. Gunnarsson, O.; Andersen, O. K.; Jepsen, O.; Zaanen, J. *Phys. Rev. B* **1989**, *39*, 1708.
14. Abragam, A.; and Bleaney, B.; *Electron Paramagnetic Resonance of Transition Metal Ions* (Clarendon, Oxford, 1970).
15. T. Moriya, Phys. Rev. 120, 91 (1960).

16. S. Carretta, J. van Slageren, T. Guidi, E. Liviotti, C. Mondelli, D. Rovai, A. Cornia, A. L. Dearden, F. Carsughi, M. Affronte, C. D. Frost, R. E. P. Winpenny, D. Gatteschi, G. Amoretti, and R. Caciuffo, Phys. Rev. B **2003** 67, 094405.
17. Liviotti, E.; Carretta, S.; Amoretti, G. J. Chem. Phys. **2002**, 117, 3361.
18. Ewings R. A., Buts A., Lee M., van Duijn J., Bustinduy I., and Perring T., Nucl. Instrum. Methods Phys. Res. Sect. A **2016**, 834, 132.
